# Task relevant autoencoding enhances machine learning for human neuroscience

**DOI:** 10.1038/s41598-024-83867-6

**Published:** 2025-01-08

**Authors:** Seyedmehdi Orouji, Vincent Taschereau-Dumouchel, Aurelio Cortese, Brian Odegaard, Cody Cushing, Mouslim Cherkaoui, Mitsuo Kawato, Hakwan Lau, Megan A. K. Peters

**Affiliations:** 1 Department of Cognitive Sciences, University of California, 2201 Social & Behavioral Sciences Gateway, Irvine, CA 92697 USA; 2 Department of Psychiatry and Addictology, Université de Montréal, Montreal, H3C 3J7 Canada; 3 Centre de Recherche de L’institut Universitaire en Santé Mentale de Montréal, Montréal, Canada; 4 ATR Computational Neuroscience Laboratories, Kyoto, 619-0288 Japan; 5 Department of Psychology, University of Florida, Gainesville, FL 32603 USA; 6 Department of Psychology, University of California Los Angeles, Los Angeles, 90095 USA; 7 RIKEN Center for Brain Science, Tokyo, Japan; 8 Center for the Neurobiology of Learning and Memory, University of California, Irvine, Irvine, CA 92697 USA

**Keywords:** Human neuroscience, Machine learning, Dimensionality reduction, Task-relevant representation, fMRI, MVPA, Autoencoder, Cognitive neuroscience, Computational neuroscience, Visual system

## Abstract

In human neuroscience, machine learning can help reveal lower-dimensional neural representations relevant to subjects’ behavior. However, state-of-the-art models typically require large datasets to train, and so are prone to overfitting on human neuroimaging data that often possess few samples but many input dimensions. Here, we capitalized on the fact that the features we seek in human neuroscience are precisely those relevant to subjects’ behavior rather than noise or other irrelevant factors. We thus developed a Task-Relevant Autoencoder via Classifier Enhancement (TRACE) designed to identify behaviorally-relevant target neural patterns. We benchmarked TRACE against a standard autoencoder and other models for two severely truncated machine learning datasets (to match the data typically available in functional magnetic resonance imaging [fMRI] data for an individual subject), then evaluated all models on fMRI data from 59 subjects who observed animals and objects. TRACE outperformed alternative models nearly unilaterally, showing up to 12% increased classification accuracy and up to 56% improvement in discovering “cleaner”, task-relevant representations. These results showcase TRACE’s potential for a wide variety of data related to human behavior.

## Introduction

In studying the human brain and human behavior, we often use machine learning methods to home in on the (ideally lower-dimensional^1–4^) representations contained in multivariate, feature-rich datasets. These data typically contain noisy, task-irrelevant signals^5–7^ that we would like to filter out using methods such as multivariate decoders^8–11^, various types of autoencoders, generative adversarial networks like InfoGAN^12^, or even principal components analysis (PCA)^13–15^. However, state-of-the-art machine learning methods typically require very large datasets to train while data for individual human subjects collected with methods such as functional magnetic resonance imaging (fMRI)^5–7^ are often severely limited in sample size^16, 17^ (i.e., have very few training exemplars compared to the dimensionality of the data). Consequently, even our best, state-of-the-art methods are susceptible to overfitting on such neuroimaging data, reducing their predictive power and utility^18–20^. What’s more, parametric methods (such as PCA), which may better avoid the need for large training sets, by definition require specific assumptions regarding the nature of the dimensionality reduction process (e.g., the common assumption of linear dimensionality reduction) and thus are limited a priori to insights consistent with these parametric assumptions. One might hope to partially alleviate the data volume issue by functionally pooling data across participants using techniques such as hyperalignment^21–24^. However, such methods can introduce other challenges stemming from domain shift between individuals (i.e., statistical differences in voxels’ response distributions across subjects); such domain shift can be particularly insidious in biological datasets such as fMRI^25^, and collecting large numbers of subjects is also burdensome financially and logistically. Thus, we are in need of a nonparametric method that can reveal the *low-dimensional, task-relevant* representations in a given brain region using *exemplar-poor but input-dimension-rich* datasets, i.e. individual subjects.

**Fig. 1:**
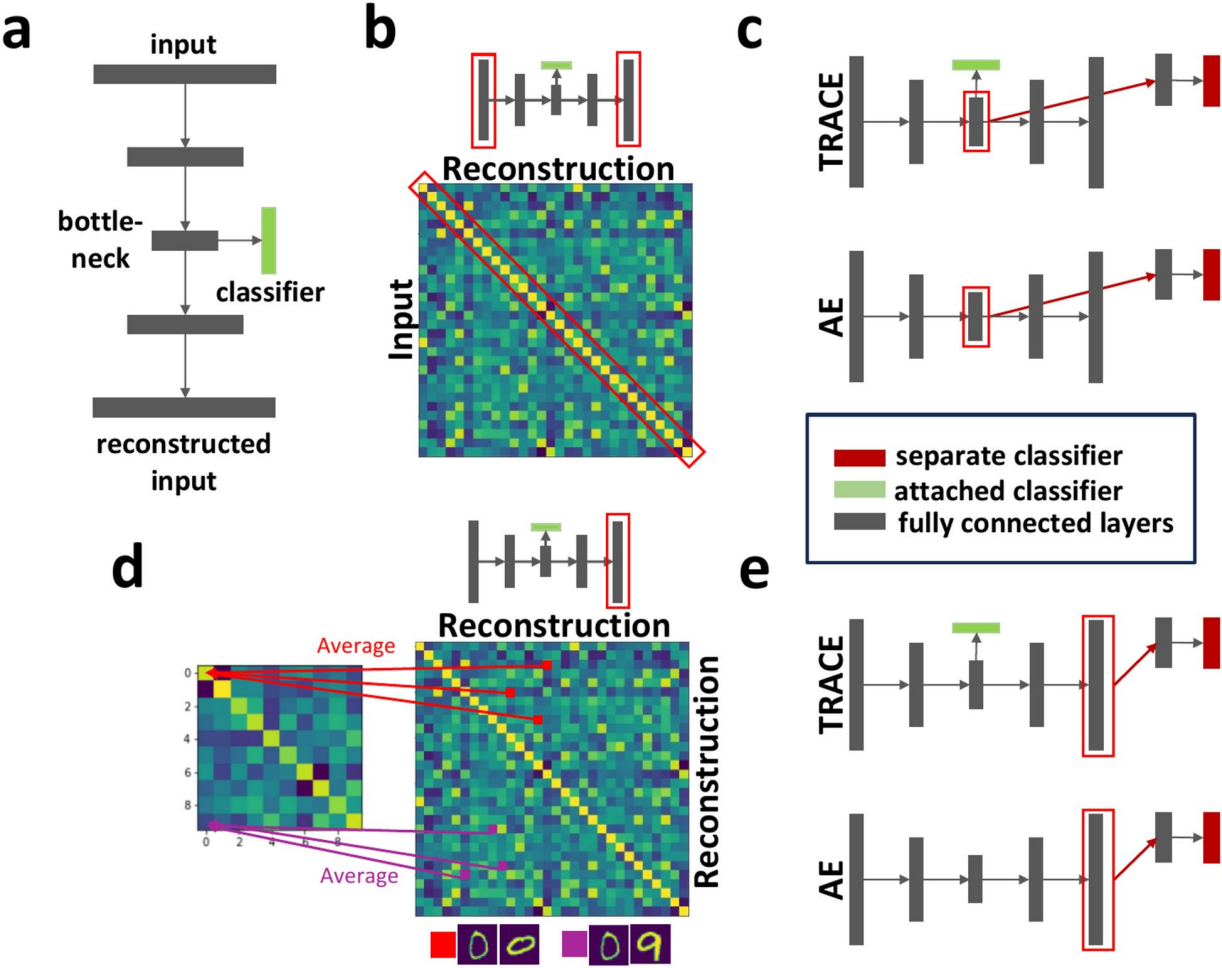
Cartoons showing the TRACE network architecture and the four evaluation metrics. (**a**) In the TRACE architecture, each gray rectangle represents a layer of the autoencoder, consisting of fully connected units. The input layer is connected to the bottleneck layer via one hidden encoding layer, and again to the reconstruction layer via one hidden decoding layer. A classifier is attached to the bottleneck and contributes to the objective optimization function. Remaining panels show the quantitative evaluation metrics: (**b**) reconstruction fidelity, (**c**) bottleneck classifier accuracy, **(d)** reconstruction class specificity, and (**e**) reconstruction classifier accuracy. Small cartoons of the TRACE architecture use red rectangle overlays to indicate which sections of the model architecture are being utilized for each outcome metric. In (**c**) and (**e**), red-filled boxes indicate separate classifiers not included when training the model, green-filled boxes indicate attached classifiers which contribute to the model’s loss function, and gray-filled boxes indicate fully-connected encoder and decoder layers. All metrics are shown with reference to TRACE and a complexity-matched standard autoencoder (AE) for simplicity, but the metrics are applied equivalently across all models; see Methods for details.

Here, we sought to capitalize on a desirable property of many human neuroimaging datasets, which is that the features we wish to identify can be conceptualized based on whether they are relevant for the subject’s behavior. We drew inspiration from previous successes with classifier-enhanced autoencoders^26–29^ to develop the Task-Relevant Autoencoder via Classifier Enhancement (TRACE) model. TRACE’s architecture is purposely simple to limit overfitting to small datasets, consisting of a fully-connected autoencoder with only one hidden layer on each of the encoding and decoding arms and a logistic regression classifier attached to the bottleneck layer (Fig. 1a).

We developed four metrics to assess TRACE’s performance at different bottleneck dimensionalities (compression levels) (Fig. 1b–e). We then exhaustively benchmarked TRACE under conditions of severe data sparsity using the popular MNIST^30^ and Fashion MNIST^31^ datasets. Having established TRACE’s efficacy, we then applied TRACE to a neuroimaging (fMRI) dataset of subjects who viewed and categorized animals and objects while blood oxygen level dependent (BOLD) signal was collected from ventral temporal cortex (VTC) in a single, 1-hour session. By constraining the dimensionality reduction process to specifically prioritize features that were relevant to the participants’ behavioral task, we show that TRACE can extract both quantitatively and qualitatively ‘cleaner’ representations at both reduced dimensions and – critically – in the original input space, showing over 12% improvement in decoding accuracy and separation of class-specific patterns. Thus, TRACE can distill highly separable, low dimensional neural representations even with sparse and noisy data, and these cleaner representations can also be projected back into the original input space. For fMRI, this could be used for understanding the nature of neural representations characterized by multivoxel patterns in original, subject-specific anatomical or functional locations. TRACE may thus show promise on a broad variety of behaviorally-relevant neuroimaging datasets.

## Results

We quantified the performance of the Task-Relevant Autoencoder via Classifier Enhancement (TRACE) model against that of a standard autoencoder (AE) and a Variational Autoencoder (VAE), and using principal component analysis (PCA) via (1) *reconstruction fidelity,* (2) *bottleneck classifier accuracy*, (3) *reconstruction class specificity,* and (4) *reconstruction classifier accuracy*, (see Methods; Fig. 1b–e) (“class” here refers to the class of the input image, e.g. “9” or “shoe” or “cat”). We assessed these metrics as a function of different bottleneck dimensionalities (i.e., compression levels), first on the MNIST and Fashion MNIST datasets under increasing data sparsity and then on a previously-collected fMRI dataset of ventral temporal cortex (VTC) (i.e., voxel activations while 59 human subjects viewed 40 classes of animals and objects). We also performed additional investigation at each dataset’s ‘optimal’ bottleneck dimensionality (where reconstruction class specificity is maximized) to characterize each model’s behavior.

### Benchmarking TRACE’s advantages, including under increasing data sparsity.

**Fig. 2:**
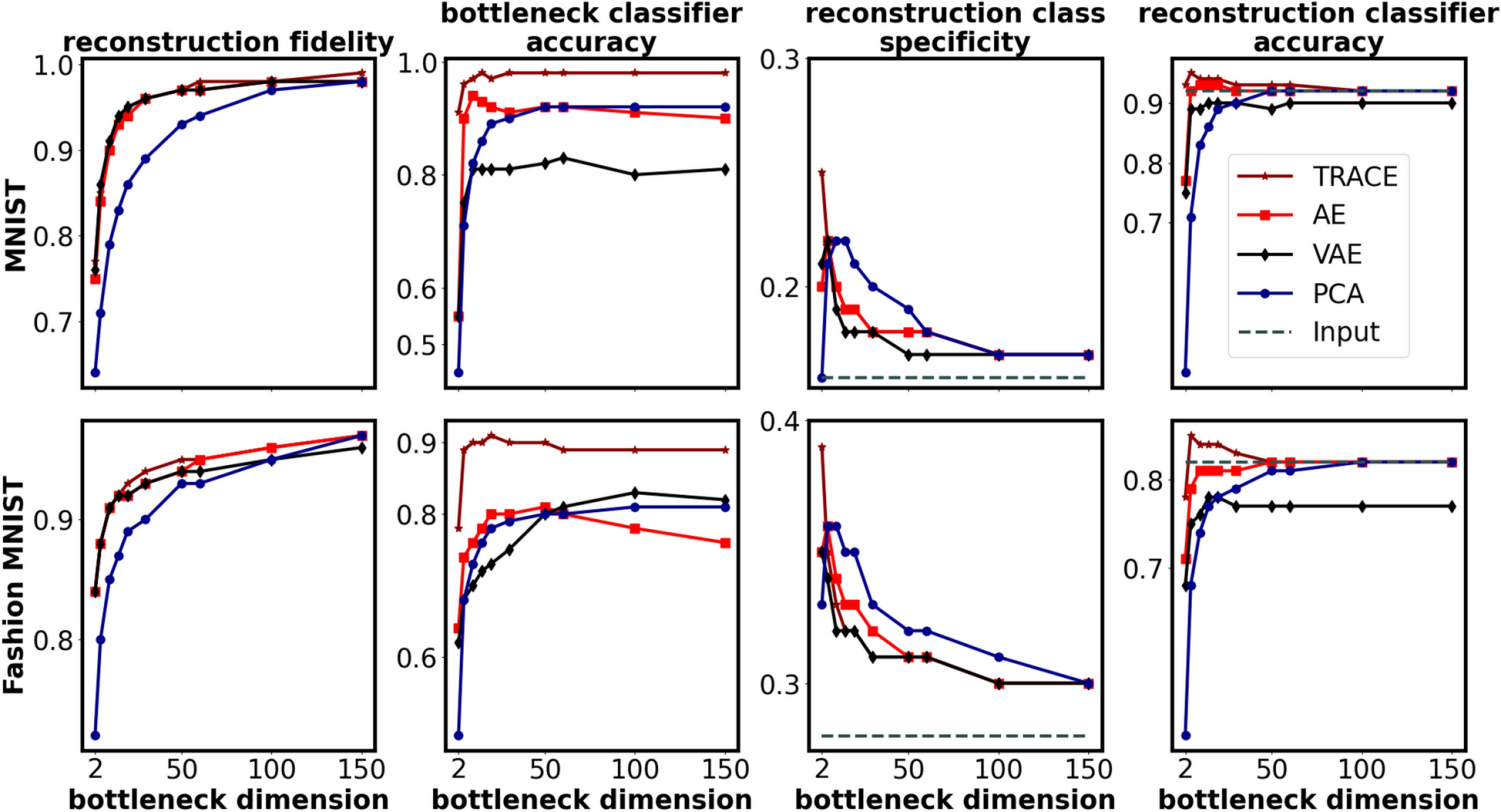
Quantitative comparison between TRACE and other models (AE, VAE, and PCA) on the four outcome metrics, for the two benchmark datasets (MNIST & Fashion MNIST) for bottleneck dimensionalities between 2 and 150. Columns show the different evaluation metrics: reconstruction fidelity, bottleneck classifier accuracy, reconstruction class specificity, and reconstruction classifier accuracy (see Methods and Fig. 1b–e). TRACE is indicated by the dark red line in all panels, with other models indicated by other colors; the dashed lines show the input class specificity and input classifier accuracy in the relevant panels. Outcome metrics for all bottleneck dimensionalities tested (dimensionalities of 2–784) are shown in Figure S2; locations of peaks for all four metrics are shown in Table S1. The chance levels of bottleneck and reconstruction classifier accuracy are both 10% (not shown in the plot). Note that conducting statistical tests is not feasible since the results reported here come from the training of cross-validated models on the entire dataset at each dimensionality of the bottleneck.

For *reconstruction fidelity* (Fig. 2, first column), TRACE performed similarly to other models despite the fact that the contribution of the reconstruction part of the loss function (mean square error; MSE) for TRACE was smaller than for AE and VAE (i.e., the objective function in TRACE is the sum of reconstruction loss (${L}_{R}$) and classification loss functions (${L}_{CE}$); see Methods).

*Bottleneck classifier accuracy* (Fig. 2, second column) was much higher for TRACE than for other models even at very low bottleneck dimensionalities. As bottleneck dimensionality grew, this metric asymptotically equalized to at least ~ 10% better than all other models in the MNIST and Fashion MNIST datasets. Notably, though, in both datasets, at all bottleneck dimensionalities tested, TRACE bottleneck classifier accuracy was *always* higher than that of other models. While attaching a cross entropy loss function to the bottleneck is of course expected to cause the network to discover features that increase the classification accuracy, it is notable here that TRACE gained this ability without losing reconstruction capacity.

*Reconstruction class specificity* (Fig. 2, third column) peaked at bottleneck dimensionality d = 2 for TRACE for both MNIST and Fashion MNIST. As with the other metrics, TRACE outperformed other models at optimal bottleneck dimensionality d = 2.

*Reconstruction classifier accuracy* (Fig. 2, fourth column) for both MNIST and Fashion MNIST peaked at bottleneck dimensionality d = 5 for TRACE, and was consistently higher for TRACE over other models. Interestingly, that this metric peaks at higher bottleneck dimensionalities than reconstruction class specificity suggests that the performance of a classifier trained on these high-dimensional reconstructions may not meaningfully reflect the maximum compression that TRACE can achieve without loss of overall performance.

A final – and critical – test of TRACE would examine its ability to not only distill task-relevant information into low-dimensional representations but also ‘push’ such distilled insights back into the native space of the input. This would be especially important if one wished to use TRACE to de-noise fMRI data to discover multivoxel patterns representing a target concept or category – e.g. to be used with noninvasive intervention strategies such as decoded neurofeedback (DecNef)^32–35^, or to simply investigate those activity patterns in native space. Although iterative sparse logistic regression and support vector machine classification have been demonstrated as successful at identifying such patterns when trained on the native input data^32, 36, 37^, we wanted to see whether TRACE would be able to denoise the data such that an even cleaner target pattern would become discoverable. Specifically, if TRACE is successful at actively removing task-irrelevant noise rather than simply passively averaging across it (as is done with input to a standard category-based logistic regression) or removing it through iterative sparsity approaches (iterative sparse logistic regression), then we should observe two patterns. First, reconstruction classifier accuracy should approach or exceed classification accuracy of an identical logistic regression classifier trained on the native inputs. Second, reconstruction class specificity should behave similarly, approaching and then exceeding input class specificity. This behavior makes reconstruction class specificity an ideal metric for defining the ‘optimal bottleneck dimensionality’ if one’s goal is to optimally distill representations in native space.

To evaluate this behavior, we (a) trained an additional logistic regression classifier on each of the datasets to classify the native input, and (b) computed class specificity directly from the raw input data for all three datasets; we then compared the outcomes to the reconstruction classifier accuracy and reconstruction class specificity computed as a function of bottleneck dimensionality (Methods). Reconstruction class specificity (Fig. 2, third column) exceeded input class specificity (dashed line) at most bottleneck dimensionalities for all models, but was higher for TRACE at the optimal bottleneck dimensionality (d = 2). Reconstruction accuracy (Fig. 2, fourth column) showed a slightly different pattern. For MNIST, reconstruction classifier accuracy exceeded input classifier accuracy (dashed line) immediately (at d = 2) for TRACE but not until d = 10 for AE; it never exceeded the input for other models. For Fashion MNIST, this occurred at d = 5 for TRACE and only at much higher dimensionality – if at all – for the other models tested. Thus, TRACE provides not only superior compression but also superior denoising even in comparison to the direct inputs.

(Note: conducting statistical tests of the results from Fig. 2 is not feasible since the results reported here come from the training of cross-validated models on the entire dataset at each dimensionality of the bottleneck.)

#### Comprehensive comparison across metrics as a function of increasing data sparsity.

We next sought to select a single bottleneck dimensionality for TRACE to explore its benefits over AE, VAE, and PCA under increasing data sparsity. We selected the optimal bottleneck dimensionality where TRACE’s reconstruction class specificity peaked, which was at d = 2 for both MNIST and Fashion MNIST (see Methods). (Recall that TRACE’s superior extraction of task-relevant information at d = 2 comes at no loss in reconstruction fidelity; Fig. 2; Table S2.) All analyses below were thus performed at bottleneck dimensionality d = 2.

To examine how TRACE fared versus the other models under increasing data sparsity, we trained each model after removing 10, 30, 50, 70, 90, 95, and 98 percent of the training data. Training examples at each level of sparsity for all models remained the same. We then used the conventional 10,000 held-out test set on the trained models and calculated all four metrics for all levels of data sparsity.

**Fig. 3:**
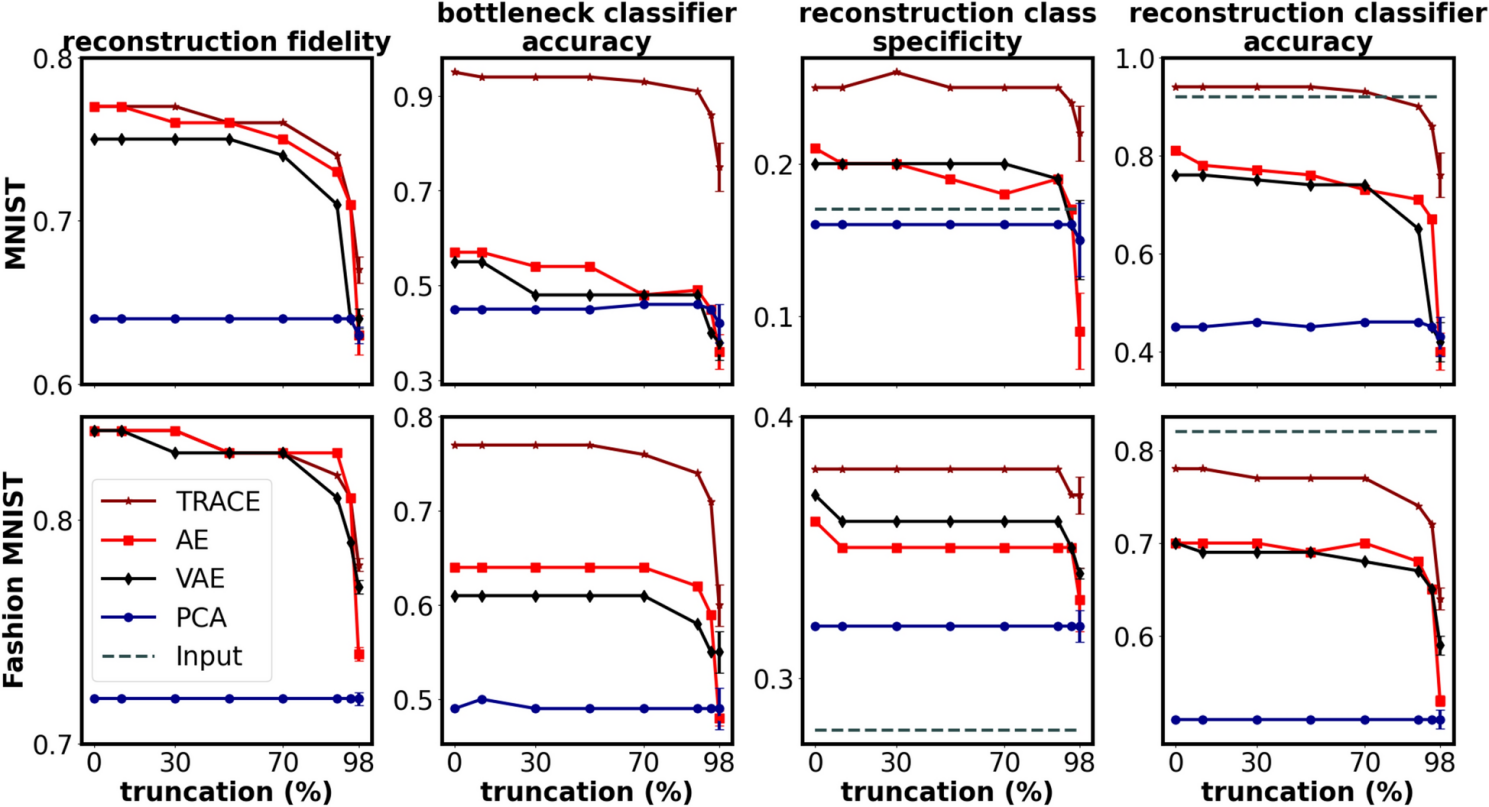
Performance of TRACE and other models as a function of sample size for the optimal bottleneck dimension of d = 2. At 98% truncation level, we used 50 independent jack-knife resamplings to truncate 98% of exemplars and reported the means and standard deviations of the metrics (calculated on the standard test set) for MNIST and Fashion MNIST. Error bars show the standard deviation of results across the 50 jack-knife resamplings at 98% data truncation. Small variations in the metrics are likely due to random initialization of weights and use of GPUs in fitting the models.

TRACE was much more robust to increasing data sparsity than other models (Fig. 3), even when only 2% of the data (1200 samples) remained available for training (matching the training conditions for the MNIST and Fashion MNIST datasets to the range of available data in the fMRI dataset). At this 98% truncation level, we performed 50 jack-knife replications to select 2% of exemplars in MNIST and Fashion MNIST for training, and reported the mean values (calculated within the standard test set) of the 50 independent training sets for all metrics.

TRACE nearly uniformly swept other models across all performance metrics (Fig. 3). We confirmed visual analysis with four one-way repeated measures ANOVAs at 98% truncation – one for each outcome metric – with factor model (4 levels). We then followed each omnibus ANOVA with planned contrasts comparing TRACE to each other model in a pairwise fashion. This analysis revealed a main effect of model for all four outcome metrics, and that TRACE was statistically superior to all other models in all 12 pairwise comparisons (see Table S3 for all statistics).

**Fig. 4:**
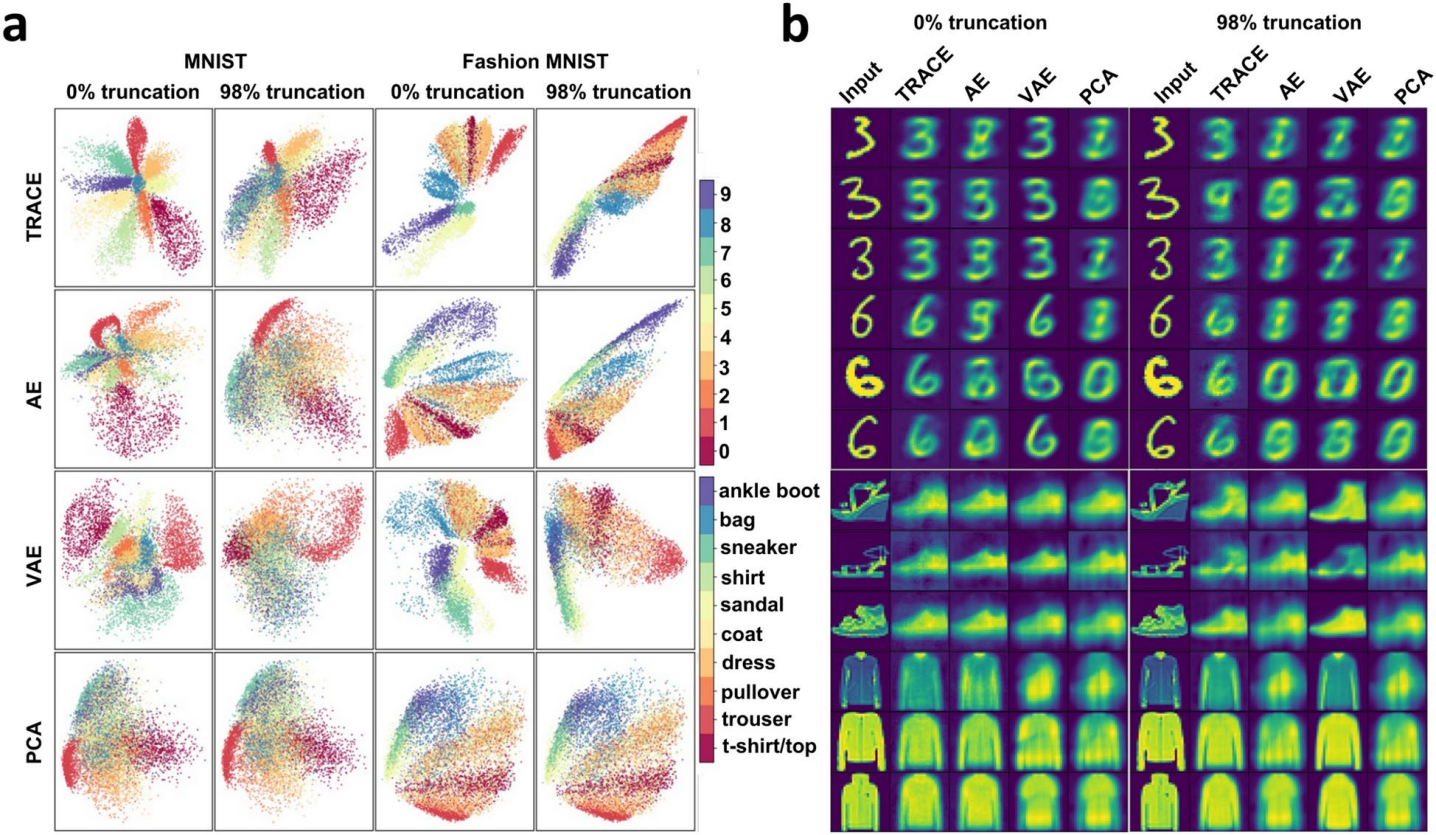
Visualization of bottleneck features and reconstructions for MNIST and Fashion MNIST datasets using TRACE, AE, VAE, and PCA. (**a**) When trained on the full dataset, TRACE shows clear superiority in creating distinctive clusters in the bottleneck for different classes for MNIST dataset in comparison to other models The distinction is less clear but still apparent in the Fashion MNIST dataset. This pattern persists even at the 98% truncation level (trained on only 2% of the data), again showing the robustness of TRACE. (**b**) The reconstruction of three representative instances of numbers “three” and “six” in MNIST dataset and three instances of classes “sandal” and “shirt” in the fashion MNIST dataset when there are two features in the bottleneck shows the same pattern. TRACE shows a more clear and *canonical* reconstruction of the inputs across several exemplars from the same category.

At maximal data reduction (98% truncation), we then performed additional explorations of both bottleneck representations and reconstructions. First, we visualized bottleneck representations by plotting the activities of the two bottleneck features against each other for each of the 10 classes in each dataset for TRACE versus the other models (Fig. 4a). The results are striking: TRACE showed superior task-relevant representations especially for MNIST, i.e. a clear qualitative advantage in clustering performance showing distinct clusters for different classes in stark contrast to the other models’ class clusters, which are heavily overlapping. Although this difference in clustering ability was less apparent for Fashion MNIST, TRACE’s clusters do appear visually more tightly bound.

We next turned to examining the reconstructions (still at bottleneck d = 2). We first examined the MNIST reconstructions for several different exemplars of the same categories (e.g., several different “3” and “6” exemplars). TRACE’s superiority is clear to the naked eye: the reconstructions of particular “3” and “6” exemplars from TRACE are much more “three-like” and “six-like” than reconstructions from other models, especially at 98% truncation (Fig. 4b). Similar findings held for Fashion MNIST (e.g., sandal and shirt), although the visual result is less striking.

We next wanted to quantitatively investigate the distributions of within-class versus between-class clusters, both in the bottleneck and the reconstructions. This approach will facilitate evaluation of the fMRI dataset since visual inspection in fMRI data is not possible in the same sense as for MNIST and Fashion MNIST given that optimal bottleneck dimensionality is larger than 2. We computed the effect size (Cohen’s d) separating clusters in both the bottleneck and reconstructions using pairwise within- versus between-class Euclidean distances (see Methods). Whether trained on all of the data or 98% truncated, Cohen’s d was always larger for TRACE than for other models (Table 1; with the one exception that they were equivalent for Fashion MNIST at 98% truncation between TRACE and VAE, even as VAE performed the worst on MNIST at 98% truncation).

**Table 1:** Cohen’s d measures of effect size comparing within-class versus between-class Euclidean distances in the bottleneck and reconstructions for the MNIST and Fashion MNIST datasets, for all models, and at 0% and 98% data truncation. Shown also are the effect size results for the control analysis, where class labels were shuffled for TRACE; see main text for details.

	0% truncation			98% truncation	
		MNIST	Fashion MNIST	MNIST	Fashion MNIST
Bottleneck	TRACE	1.63 ± 0.21	1.65 ± 0.5	1.36 ± 0.41	1.45 ± 0.35
	AE	1.1 ± 0.39	1.5 ± 0.43	1.01 ± 0.41	1.26 ± 0.44
	VAE	1.32 ± 0.55	1.61 ± 0.51	0.8 ± 0.31	1.48 ± 0.44
	PCA	1.06 ± 0.54	1.25 ± 0.59	1.06 ± 0.54	1.25 ± 0.59
Reconstruction	TRACE	1.58 ± 0.21	1.6 ± 0.69	1.51 ± 0.63	1.54 ± 0.68
	AE	1.2 ± 0.29	1.48 ± 0.62	1.02 ± 0.44	1.41 ± 0.66
	VAE	1.27 ± 0.28	1.42 ± 0.61	0.68 ± 0.38	1.37 ± 0.65
	PCA	1.06 ± 0.54	1.25 ± 0.59	1.06 ± 0.54	1.25 ± 0.59
TRACE trained and evaluated with shuffled labels					
Bottleneck		0.002 ± 0.004	0.003 ± 0.005	0.001 ± 0.008	0.002 ± 0.0.002
Reconstruction		0.004 ± 0.003	0.005 ± 0.008	0.004 ± 0.010	0.003 ± 0.001

Finally, we conducted a control analysis to ensure that the TRACE model does not manufacture or impose structure where none truly exists by randomizing the category labels across exemplars before training and evaluating TRACE at d = 2, both for 100% of the available training data and at 98% truncation (see Methods). Cohen’s d analysis confirmed that with shuffled labels, TRACE does not ‘discover’ or hallucinate spurious patterns, since Cohen’s d for TRACE with shuffled labels was essentially 0, for both the 0% and 98% truncation levels (Table 1).

### TRACE’s performance on a real fMRI dataset

We next evaluated TRACE in comparison to the other models using a real-world fMRI dataset, since ultimately our goal is to learn about neural representations. Thus, we used the same metrics as we used to evaluate TRACE on MNIST and Fashion MNIST on an fMRI dataset consisting of 59 individuals who each viewed 3600 exemplars of 40 classes of animals and objects (90 exemplars per class) while BOLD signal from ventral temporal cortex (VTC) was obtained. The number of voxels in VTC for each individual was different; however, the average of voxels for the 59 subjects was 2382 $\pm$ 303.

**Fig. 5:**
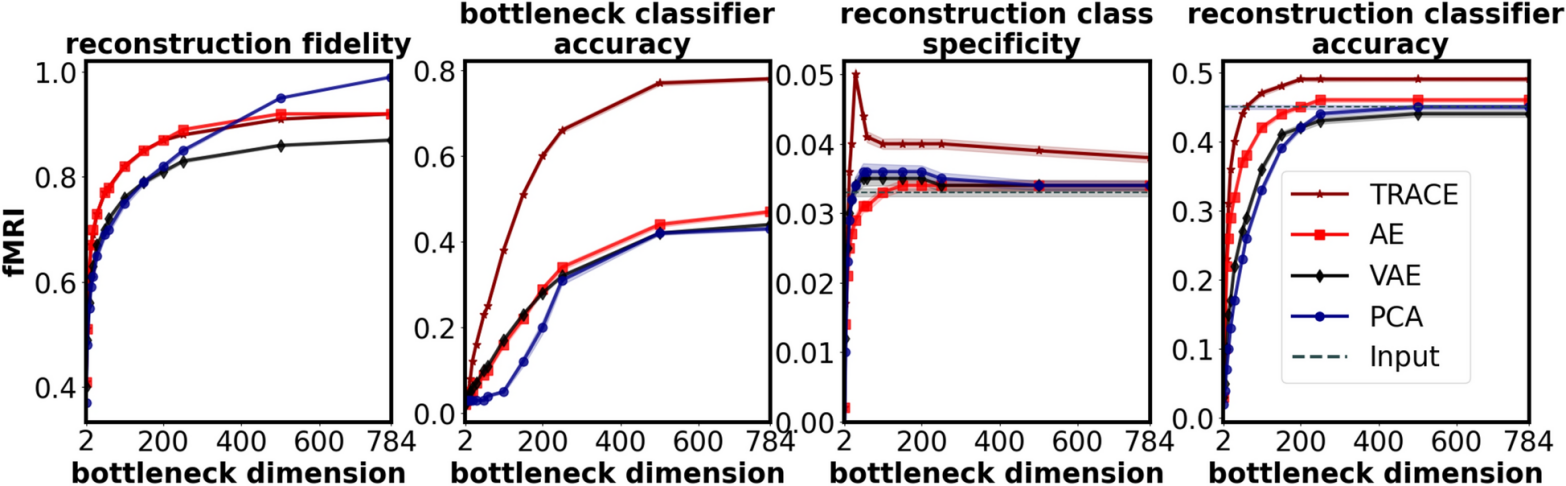
Comparison between quantitative metrics for TRACE and other models for fMRI dataset (n = 59). TRACE shows superior performance in three out of four metrics (excluding reconstruction fidelity and only for d > 250).

Excitingly, the fMRI dataset showed largely the same patterns as the MNIST and Fashion MNIST datasets (Fig. 5). First, while reconstruction fidelity was actually slightly higher for AE over TRACE and VAE at higher dimensions, note that the numerical difference between TRACE and AE is very small, and that both are outperforming VAE; AE’s slight superiority is likely due to the fact that reconstructing the input is AE’s only objective. PCA also showed higher reconstruction fidelity than all other models starting around d = 500, which is also expected since as the number of principal components increases, the PCA model can explain the variance of the input data almost perfectly. TRACE also showed higher bottleneck classifier accuracy at all bottleneck dimensionalities in comparison to other models.

TRACE outperformed other models in reconstruction class specificity as well, showing that even in the native space of the input – i.e., voxel patterns of activity in ventral temporal cortex – TRACE not only successfully distills lower-dimensional representations of task-relevant data, but also faithfully projects them back into original, high-dimensional voxel space. Reconstruction class specificity peaked at bottleneck dimensionality d = 30, and then fell again. The same was not true for other models, for which reconstruction class specificity rose but then largely asymptoted. Crucially, though, reconstruction class specificity was also always higher for TRACE than for other models, much exceeding input class specificity. This capacity to distill a task-relevant, low-dimensional representation and put it back in brain space could potentially have great value for studies in which such multivoxel patterns are the target of DecNef^32–35^ or other investigations which may desire the flexibility or spatial interpretability of anatomically-related representations rather than a latent space embedding. We discuss this possibility in greater detail in the Discussion, below.

Finally, TRACE’s reconstruction classifier accuracy asymptoted at bottleneck dimensionality around d = 250 for all models, but again TRACE showed higher reconstruction classifier accuracy than AE, VAE, and PCA at all bottleneck dimensionalities tested. Notably, reconstruction classifier accuracy even surpassed the input classifier accuracy for bottleneck dimensionalities higher than d = 60 which again suggests that the reconstructed version in the original input space contains more task-relevant information than the original input.

#### Exploration at optimal bottleneck dimensionality for fMRI data.

As mentioned above, the maximal value for reconstruction class specificity was found at d = 2 for the MNIST and Fashion MNIST datasets. For the fMRI dataset, we found that reconstruction class specificity peaked at d = 30, so we proceeded with a parallel analysis to that done above at this dimensionality.

Crucially, at d = 30, TRACE’s performance on the fMRI dataset mimicked its exemplary performance on the MNIST and Fashion MNIST datasets with the exception of reconstruction fidelity, which was only slightly smaller for TRACE than for AE at dimensionalities of d > 250 (and PCA at higher dimensions [i.e., d > 500]) (Fig. 5). To quantify this superiority, we performed a one-way repeated measures ANOVA for each outcome metric with factor model (4 levels), followed by planned pairwise contrasts comparing TRACE to every other model. Results revealed significant main effects of model for all four outcome metrics, and that TRACE outperformed the other models in 11 of these planned comparisons (with the exception of reconstruction fidelity between TRACE and AE; see Table S4 for statistics).

Ultimately, as our goal is to learn about representations in human VTC, we also might want to visualize clusters for the 40 classes of the fMRI dataset. However, unlike for MNIST and Fashion MNIST where optimal bottleneck dimensionality was d = 2, for the fMRI dataset we found the optimal bottleneck dimensionality at d = 30. Therefore, we cannot easily visualize the class clusters in a scatterplot, and performing further dimensionality reduction for the sake of visualization would be inappropriate since assumptions of whichever dimensionality reduction technique we chose would impact the visualizations. Instead, we can use the Cohen’s d approach described above to characterize the tightness of the class clusters even in higher dimensionalities. The average effect size separating within- and between-class Euclidean distances across all 59 subjects was 0.38 (± 0.09) for TRACE, 0.12 (± 0.03) for AE, 0.11 (± 0.02) for VAE, and 0.08 (± 0.02) for PCA, again showing TRACE’s superiority.

As a final evaluation of TRACE’s ability to filter out task-irrelevant information, we calculated the within- versus between-class Euclidean distance Cohen’s d in the reconstructions. Note that visually examining fMRI reconstructions would not provide particularly useful information about the ‘cleanliness’ of the reconstruction, as the patterns are not visually meaningful to begin with, so we must again rely on a quantitative comparison. The average Cohen’s d here again showed TRACE’s superiority, with mean Cohen’s d of 0.14 (± 0.02) across subjects for TRACE, 0.09 (± 0.02) for AE, 0.08 (± 0.02) for VAE, and 0.08 (± 0.02) for PCA. In other words, TRACE was able to reduce task-irrelevant information and thus extract a ‘cleaner’ representation, even in the reconstructions.

Finally, to ensure that the TRACE model does not ‘hallucinate’ structure where none truly exists also in the fMRI data, we again randomized the category labels across exemplars before training and evaluating TRACE on the fMRI data at d = 30 (see Methods). This analysis revealed that with randomized labels, TRACE’s average Cohen’s d effect size across subjects for the bottleneck was 0.003 (± 0.071), while for reconstruction it was -0.003 (± 0.063) – essentially, at 0. Thus, compared to TRACE’s effect sizes achieved with correct labeling, the shuffled label control analysis shows that TRACE is unlikely to impose structure where it does not exist.

## Discussion

### Summary of findings

In neuroimaging research, we often wish to identify clean, generalizable patterns of neural activity – whether our goal is basic science understanding or clinical application. Unfortunately, many state-of-the-art deep learning models are of limited utility for discovering and characterizing meaningful representations in input-dimension-rich but exemplar-poor datasets, as they tend to overfit^18–20^. Further, many approaches also do not have a specific mechanism to ensure that the representations they reveal are particularly relevant to the target mental representations of interest rather than task-irrelevant features. Together, these facts make extracting neural representations in within-subject fMRI datasets – which often contain a high degree of noise and task-irrelevant information – extremely challenging^5–7^.

To address these issues we proposed the TRACE model: a simple autoencoder with a classifier attached to the bottleneck. The classifier forces TRACE to learn not just lower dimensional representations of the data, but those that are also task-relevant – and thus, maximally relevant to the scientific questions for which a particular study was likely designed. To quantify TRACE’s superiority over a standard autoencoder (AE), a variational autoencoder (VAE), and principal components analysis (PCA), we used four metrics (see Methods): 1. reconstruction fidelity; 2. bottleneck classifier accuracy; 3. reconstruction class specificity; and 4. reconstruction classifier accuracy.

TRACE outperformed all other models in all metrics, with the exception of reconstruction fidelity (sometimes). Moreover, at the ‘optimal’ bottleneck dimensionality, TRACE’s superiority was evident in both the bottleneck and reconstruction, and TRACE’s reconstructions could even outperform the inputs on measures of task-relevant behavior (reconstruction class specificity and reconstruction classifier accuracy). TRACE’s advantage over other models appears due to its capacity to minimize task-irrelevant, idiosyncratic information unique to a particular sample of a target behaviorally-relevant class. This is evident in the one occasional exception to TRACE’s sweeping superiority: its slight loss to AE for fMRI reconstruction fidelity. However, this seeming underperformance is actually a strength, since AE tried “too hard” to encode idiosyncratic details of a particular exemplar in the bottleneck. Some of those details were merely noise for the task that the observer is performing, and precise reconstruction of noise is undesirable.

Critically, TRACE outperformed all other models even under extreme data truncation for the MNIST and Fashion MNIST datasets and in a real-world fMRI dataset, showing that TRACE can succeed in discovering generalizable patterns even when there is a highly undesirable balance of input-dimensions versus samples. Since sample scarcity is typical in fMRI data, TRACE’s superiority suggests its strong promise even beyond fMRI datasets for other biological-scale data with many more input dimensions than samples^25^.

### Relation to previous work

Our approach builds on previous successes with classifier-enhanced autoencoders^26–29^ to extract task-relevant representations in non-biological datasets such as linguistic datasets, standard computer vision object datasets, and fault diagnosis applications. However, TRACE goes beyond these previous successes by explicitly demonstrating with otherwise matched architecture (TRACE vs AE) that the simple addition of a classifier can improve extraction of task-relevant latent representations *even under extreme data paucity*. This demonstration is especially important for the types of data used in cognitive neuroscience, which are often sample-poor. We also demonstrate that TRACE can improve reconstruction classifier accuracy and reconstruction class specificity such that it exceeds even input-level for these metrics, which could be a boon for DecNef^32, 33, 38^. We discuss these implications in more detail below.

Other techniques have been developed including nonparametric techniques beyond the fully-connected AE and VAE^39, 40^ used here^41, 42^: adversarial autoencoders^43^, generative adversarial networks (GANs)^44^, deep convolutional GANs (DCGANs)^45^, and so on. While comprehensive exploration of these is beyond the present scope, we note that many of these models do still suffer from the fact that the discovered lower dimensional representations are not explicitly crafted to be task-relevant^46^. To drive this message home, we additionally show that a conditional GAN (a GAN modified to allow selection of specific categories of reconstruction;^47^) fails quite miserably when trained on only 2% of the MNIST or Fashion MNIST datasets (Supplement S3, Figure S4). These models’ lack of task-relevance led to the development of InfoGAN^12^, an unsupervised learning technique which modifies a generative adversarial network (GAN) in order to learn interpretable, low-dimensional representations. InfoGAN accomplishes this task by maximizing mutual information between noise in the GAN network and observations. Yet despite the tremendous success of InfoGAN^12^, it is highly disadvantaged for the limited (sample-poor) data type targeted here. Specifically, InfoGAN’s success has been demonstrated only on large-scale training datasets consisting of tens of thousands of training images. Further, exploring and characterizing latent spaces in GANs in general is highly nontrivial^48, 49^; for these reasons, GANs generally do not accomplish the goal targeted by the TRACE network.

Attempts to mitigate the curse of dimensionality in fMRI datasets by pooling data across subjects to create larger training sets have of course been established to try to mitigate this significant challenge, including the shared response model^4^, hyperalignment^21, 24, 50^, and more recently decoder + autoencoder approaches^51^. However, while these can pool fMRI data to create more training samples, they do not explicitly seek subject-specific response patterns and instead presuppose that all subjects share a common response pattern to at least some extent. Addressing such individual differences in patterns is a major domain adaptation or transfer learning challenge in its own right, the difficulty of which is exacerbated by the nature of fMRI data (small datasets, feature complexity, and so on); see^25^ for further discussion.

In sum, although we do not benchmark TRACE against InfoGAN, hyperaligned data, or the expansive space of model variants, we argue that TRACE’s utility is not only in its ability to distill task-relevant, low-dimensional representations, but also to do so in exemplar-limited, biological-scale datasets such as those collected in human neuroimaging experiments within a single subject.

### Limitations

One limitation of the present approach is that we (deliberately) made TRACE and other models extremely simple (as in, few layers), which could have limited their performance. We did not investigate whether TRACE-like architecture (addition of a classifier on the bottleneck layer) would similarly improve performance for more complex networks, or whether multi-layer perceptrons or convolutional neural network (CNN) classifiers would surpass the simple logistic regression classifiers used here. We also could have opted to make the models deeper, with many hidden layers, which might have resulted in benefits in classification or reconstruction. However, we reiterate that we selected a simple architecture to be able to best evaluate TRACE’s advantages over a “plain vanilla” fully-connected autoencoder, as more complex architectures could obscure TRACE’s advantages. Future work may wish to explore other possible TRACE-like architectures.

It is also worth mentioning that for the sake of consistency we kept all hyperparameters for all networks and datasets the same. However, during training TRACE on a new dataset, it is always possible to tune the hyperparameters (learning rate, batch size, regularization, etc.) in order to achieve better performance (e.g. better bottleneck classification accuracy). Future studies may also more comprehensively explore the impact of specific hyperparameter tuning choices on TRACE’s behavior.

Finally, it is worth noting that we did not comprehensively explore TRACE’s specific denoising capacities, in that we did not seek to impose noise into the training data and then remove it. The structure of noise, and how it relates to underlying signal patterns, is indeed of critical importance to the performance of any TRACE-like architecture for denoising purposes. As our primary goal here was both to examine denoising capacities but also to evaluate TRACE’s ability to extract lower-dimensional representations under conditions of data sparsity, a comprehensive exploration of the denoising capacities of TRACE or similar models under various biologically-plausible and modality-dependent noise characteristics is beyond the scope of the present project. Indeed, though, exploration of the specific idiosyncracies of biological datasets and how they interact with machine learning modeling approaches is of critical importance for future work, as it is known that the specific characteristics of biological data (in terms of feature types, feature importances, and so on) may make them challenging to work with using even state of the art models^25^. We therefore leave these exciting explorations to future studies.

### Implications & future directions

Our findings have potentially exciting implications for the discovery of both low-dimensional representations and representations in the original (and anatomically- and/or functionally-relevant, in the case of fMRI) input space. For example, if a study’s goal is to induce canonical target patterns of neural activity for a particular object category with DecNef^32, 33, 38^, one might also wish to evaluate the empirical benefit of replacing maximizing reconstruction class specificity with maximizing reconstruction classifier accuracy for selecting the optimal bottleneck dimensionality. In the fMRI dataset presented here, reconstruction classifier accuracy peaked at about d = 200. It is possible that in other fMRI datasets, reconstruction classifier accuracy might peak at a non-maximal bottleneck dimensionality, in which case it could be used to select the best dimensionality for the task at hand. Alternatively, one could choose to select optimal bottleneck dimensionality based on when reconstruction class specificity or classifier accuracy exceeds the analogous metric calculated directly from the raw input data. Here we showed that TRACE either exceeds these benchmarks sooner than other models, or does so even when other models do not. Thus, the process for selecting the best bottleneck dimensionality can flexibly adapt to an experimenter’s goals, and future research seeking to use TRACE to extract neural patterns for use with DecNef should explore how different bottleneck dimensionalities impact the success of the neurofeedback process.

Regardless of the method one uses to select bottleneck dimensionality, it seems likely that TRACE can remove task-irrelevant information in a way that is useful for DecNef. To demonstrate this possibility, we did one final exploratory test. Recall that the fMRI dataset used in this study is in part overlapping with the dataset used by Taschereau-Dumouchel and colleagues^38^, and as such we can directly compare their binary (“cat” versus “everything that is not a cat”) decoding accuracy with the decoding accuracy we achieved on TRACE’s reconstructions. To translate the reconstruction classifier accuracy we achieved to a binary scale, we counted a prediction to be correct if the correct class was in the top 20 (out of 40) of predicted classes from our one-versus-all classifier (with chance classification accuracy at 2.5%). Taschereau-Dumouchel and colleagues^38^ observed binary logistic regression classification accuracies of 71.7% on average within-subject (~ 1 hour of fMRI data per person). (Relying on hyperalignment^21^ to pool their 30 subjects and subsequently train such classifiers, they observed mean 82.4% using a 30-subject concatenated dataset.) When we trained logistic regression classifiers on each individual subject (i.e., no hyperalignment) – some of whom are actually the original subjects in that former study – and translated the classification accuracies as described to be on the same scale as binary classification, we achieved the equivalent of 94.4% binary accuracy at bottleneck dimensionality d = 30 (where reconstruction class specificity was maximized). Thus, TRACE facilitates distillation of class-specific representations in native space that are superior to the original representations themselves for this purpose.

Another interesting future possibility would be to investigate the extent to which TRACE excels over other methods as a function of neuroanatomical area – for the purposes of DecNef or simply to investigate neural representations themselves. Here, we focused on object representations in high level visual cortex (VTC), but in theory one could ask how early in the visual processing pipeline we might find evidence that task relevance plays a meaningful role. In the fMRI dataset used here, the task was for subjects to identify the object category of the image, and as a result the images were not standardized across lower level visual features such that object category did indeed covary with lower level visual properties such as color or spatial frequency (e.g. the background color of the ‘dolphin’ images is predominantly blue, whereas this is not the case for the ‘key’ images). Future studies may wish to use standardized images to investigate to what extent TRACE may assist in extraction of task-relevant representations versus low-level visual properties, depending on task and brain area; due to the limitations of the dataset used here for this first proof of concept, we leave these questions to future investigations.

Given TRACE’s success here, we hope that its capacity to discover task-relevant information *despite* undesirable ratios of samples to input-dimensions can help discover truths about other biological processes. Future studies should apply TRACE to other biological-scale datasets, with the goal of discovering representations relevant to those researchers and domains.

As discussed earlier, discovering lower dimensional representations that are in fact more task relevant can greatly help researchers to interrogate these lower dimensions. It is important to acknowledge that utilizing deep learning models such as TRACE comes with the caveat of a more difficult interpretation. Thus, full exploration of the latent, low-dimensional representations extracted by TRACE remains a subject for further investigations using available explainable artificial intelligence methods^52^.

## Methods

### Methods overview

We proposed the “Task-Relevant Autoencoder via Classifier Enhancement” (TRACE) model and directly compared its behavior to that of a standard autoencoder (AE) and a variational autoencoder (VAE) with equivalent internal architecture, as well as to principal component analysis (PCA). Additional information about the methodology can be found in Supplement S1.

### Datasets

We employed the MNIST^30^ and Fashion MNIST^31^ datasets to benchmark TRACE against other models. Additionally, we used a previously collected fMRI dataset, partially reported by Taschereau-Dumouchel and colleagues^38^, in order to demonstrate the TRACE’s efficacy in small-scale fMRI datasets. The fMRI data used in this study was obtained from 59 healthy individuals who viewed 3600 images from 40 different categories of objects (30 animals and 10 man-made objects) while the whole-brain BOLD responses were acquired. See Supplement S1.2 for more information.

### Models

We developed TRACE and benchmarked it against three other models: a standard autoencoder (AE), a variational autoencoder (VAE), and standard principal components analysis (PCA). For brevity, here we introduce TRACE and briefly mention the comparison models. See Supplement S1.3 for more details.

#### Task-relevant autoencoder via classifier enhancement (TRACE)

The Task-Relevant Autoencoder via Classifier Enhancement (TRACE) model is almost identical to a standard autoencoder with two hidden layers (one in the encoding section and one in the decoding section), but also includes a logistic regression classifier attached to the bottleneck (Fig. 1a). We used the hyperbolic tangent as the hidden layers’ activation function, which is ideal for capturing detailed and local information to represent data via lower dimensions^53^. The “decoder branch” activation function was the softmax function (Boltzmann distribution), which outputs a probability distribution for each class.

The objective function of TRACE consists of two components. The first (Eq. 1) adopts the mean square error (MSE) as the criterion to reconstruct the input:

1 $${L}_{R} = \frac{1}{m\times n} {\sum }_{i=1}^{m}{\sum }_{j=1}^{n}( {\widehat{X}}_{ij}- {X}_{ij}{)}^{2}$$

where $X$ is the input with $m$ samples and $n$ input dimensions, and $\widehat{X}$ is the reconstruction.

The second component (attached to the bottleneck of the network, Eq. 2) is the cross-entropy loss function to find lower-dimensional representations optimized to be task-relevant:

2 $${L}_{CE}=\frac{-1}{m }{\sum }_{i=1}^{m}{\sum }_{c=1}^{k}{y}_{ci} log({\widehat{y}}_{ci})$$

where $k$ denotes the number of the classes, $y$ is the label of observation, and $\widehat{y}$ is the predicted label.

TRACE’s final objective function is the weighted summation of reconstruction loss and the categorical cross-entropy loss function (Eqs. 1 & 2), i.e.:

3 $${L}_{TRACE}={L}_{R}+\alpha{L}_{CE}= \frac{1}{m\times n} {\sum }_{i=1}^{m}{\sum }_{j=1}^{n}( {\widehat{X}}_{ij}- {X}_{ij}{)}^{2}-\frac{\alpha }{m }{\sum }_{i=1}^{m}{\sum }_{c=1}^{k}{y}_{ci} log({\widehat{y}}_{ci})$$

where α sets the weight for the classifier part of the loss function in order to control for its participation in updating the parameters. Details about implementation of TRACE, including optimization function and parameter settings, can be found in Supplement S1.3.

#### Other models

We compared TRACE’s behavior to that of standard autoencoder (AE), a Variational Autoencoder (VAE), and principal component analysis (PCA). Details of these comparison models can be found in Supplement S1.3.

#### Outcome metrics

We evaluated models using four metrics (Fig. 1b–e): (1) *reconstruction fidelity,* (2) *bottleneck classifier accuracy*, (3) *reconstruction class specificity*, and (4) *reconstruction classifier accuracy*. We computed these metrics for all models and datasets as a function of bottleneck dimensionalities 2, 5, 10, 15, 20, 30, 50, 60, 100, 150, 200, 250, 500, and 784 (the maximum size of the MNIST and Fashion MNIST datasets).

#### Reconstruction fidelity

We quantified models’ capacity to reconstruct inputs using the average of all Pearson correlation coefficients between each input trial of the test set and the corresponding reconstruction of that sample (Fig. 1b):

6 $${fidelity}_{R}=E({\rho }_{R})$$

where ${\rho }_{R}$ is the correlation between each input exemplar and its reconstruction, and $E$ denotes the expected value. High reconstruction fidelity assures us that the discovered features in the bottleneck provide a reasonable representation of the original high-dimensional information.

#### Bottleneck classifier accuracy

We quantified the task-relevance of bottleneck features via the accuracy of a logistic regression classifier with such bottleneck node activity as inputs (Fig. 1c):

7 $${L}_{bottleneck classifier}=\frac{1 }{m }{\sum }_{i=1}^{m}{\sum }_{c=1}^{k}{y}_{ci} log({\widehat{y}}_{ci})+\lambda {\sum }_{b=1}^{q}{{w}_{b}^{2}}$$

where $m$ is number of samples, $k$ is number of classes, $y$ is the observation label, and $\widehat{y}$ is the predicted label. Here, $\lambda$ is the regularization parameter, and $w$ and $q$ are the weight matrices and the number of parameters of the classifier respectively. For all models, this classifier is trained separately, after the training of the main model has finished.

#### Reconstruction class specificity

To quantify how well the reconstructed input represents a certain class, we can compute the degree of similarity of representations within a class versus between classes. We quantified this “reconstruction class specificity” (RCS) as the average of the diagonal (within class) of this similarity matrix minus the average of the off-diagonal (between class) of this matrix (Fig. 1d), i.e.

8 $$RCS=E({\rho }_{R,within}) -E({\rho }_{R,between})$$

where ${\rho }_{R,within}$ and ${\rho }_{R,between}$ are the Pearson correlation matrices between trials within-class and between-class, respectively.

#### Reconstruction classifier accuracy

The task-relevancy of reconstructed information can also be measured by training a separate logistic classifier using reconstructed inputs for all dimensions in the bottleneck (Fig. 1e):

9 $${L}_{reconstruction accuracy}=\frac{1 }{m }{\sum }_{i=1}^{m}{\sum }_{c=1}^{k}{y}_{ci} log({\widehat{y}}_{ci})+\lambda {\sum }_{r=1}^{p}{{w}_{r}^{2}}$$

where $m$, $k$, $y$, $\widehat{y}$, $\lambda$, and $w$ are defined as in **Eq. **7, and $p$ is the number of parameters of the reconstruction-based classifier.

#### Benchmarks against original inputs

If one wished to use TRACE to de-noise fMRI data to discover multivoxel patterns representing a target concept or category to be used with DecNef^32–35^, or to simply investigate those activity patterns in native space, superiority in a direct comparison to the ‘input’ representations would also be desirable. To quantify reduction in noise and success of task-relevant feature extraction, we thus benchmarked the reconstructions from all models in two ways.

First, we compared reconstruction class specificity to class specificity calculated using Eq. 8 on the input data rather than the reconstructions. Second, we compared reconstruction classifier accuracy (Eq. 9) to accuracy of an identical classifier trained directly on the input data. If a representation has been successfully de-noised, then the reduction in task-irrelevant noise should be apparent in superior reconstruction compared to input classification accuracy.

### Increasing data sparsity through dataset truncation

Because fMRI data are much more feature-rich and sample-poor than traditional machine learning datasets, we wish to understand TRACE’s performance also under increasing data sparsity. This evaluation requires that we first choose a single bottleneck dimensionality for TRACE to explore its benefits over AE, VAE, and PCA. For this purpose, we selected the maximal value of TRACE reconstruction class specificity because this metric provides the best balance between task-relevant information extraction and compression, both for analyzing low-dimensional representations and patterns in the original input dimensionality.

We then evaluated all models as we iteratively removed 10, 30, 50, 70, 90, 95, and 98% of the training data. At 98% truncation, the MNIST and Fashion MNIST datasets possess approximately the same samples-to-input-dimensions ratio as in the fMRI dataset used here (~ 1.6 for MNIST and Fashion MNIST, and ~ 1.5 for this fMRI dataset). We used the same training examples at each level of sparsity for all models so as to provide the most direct model comparison. We then used the conventional 10,000 held-out test set and calculated all four outcome metrics for all levels of data sparsity.

### Cohen’s d effect size

For both the bottleneck representations and reconstructions, we computed the effect size (Cohen’s d) separating clusters using pairwise within- versus between-class Euclidean distances:

10 $${d}_{c}=\frac{{\overline{D} }_{w,c}-{\overline{D} }_{b}}{s}$$

where ${d}_{c}$ is the Cohen’s d for category $c$,  ${\overline{D} }_{w,c}$ is the average pairwise Euclidean distance between exemplars in category $c$, ${\overline{D} }_{b}$ is the average pairwise Euclidean distance between exemplars in category $c$ and all exemplars in all other categories, and $s$ is the pooled standard deviation.

### Control analysis

To ensure that the TRACE model does not “hallucinate” structure (i.e., discover structure where none exists), we undertook a control analysis. This analysis involved randomizing the category labels on the training samples prior to training the TRACE model, such that there would be no meaningful structure in the input. We then evaluated TRACE by computing the Cohen’s d metrics, also with shuffled labels for the test dataset. Note that this control analysis cannot be performed on the models other than TRACE, as they do not consider any labels in their loss functions and therefore scrambling the labels would have no impact.

## Supplementary Information

Supplementary Information.

## Data Availability

The data for this project are available from the corresponding authors upon reasonable request. The code implementing all models, including outcome metrics, is available at https://github.com/mehdi-or/TRACE/.
