# Supplementary material for: Task relevant autoencoding enhances machine learning for human neuroscience
Source: Sci Rep. 2025 Jan 8;15:1365. doi: 10.1038/s41598-024-83867-6 (PMC11711280; doi:10.1038/s41598-024-83867-6)
Supplement: Supplementary file 1 — Supplementary Information. [file 41598_2024_83867_MOESM1_ESM.docx]

# “Task-relevant autoencoding” enhances machine learning for human neuroscience

**Authors:** Seyedmehdi Orouji, Vincent Taschereau-Dumouchel, Aurelio Cortese, Brian Odegaard, Cody Cushing, Mouslim Cherkaoui, Mitsuo Kawato, Hakwan Lau, & Megan A. K. Peters

# Supplementary Material

## S1 Methods and model implementation

### S1.1 Method overview

We developed TRACE and used three datasets to benchmark it against three other models: a standard autoencoder (AE), a variational autoencoder (VAE), and standard principal components analysis (PCA). TRACE is equivalent to the AE model in every respect, with the exception of a classifier branch which reads out directly from the AE network’s bottleneck layer and which contributes to the overall loss function of the network. Likewise, VAE’s architecture was chosen to be identical to AE except an additional KL-divergence loss function to make sure the features in the bottleneck followed a standard Gaussian distribution. We benchmarked TRACE and other models on the MNIST and Fashion MNIST datasets often used in machine learning model evaluation, and then applied both models to a previously-collected fMRI dataset to showcase TRACE’s utility in extracting task-relevant low dimensional representations in a nonparametric regime.

Details of datasets and model architectures are described in the next sections.

### S1.2 Datasets

#### S1.2.1 MNIST dataset

The MNIST dataset consists of 60,000 handwritten 28x28 pixel grayscale images (i.e., image dimensionality of 784 pixels) of the 10 classes of digits (i.e., 0,1,...9) with their corresponding labels as the training set and 10,000 samples as the test set with a total of 7000 images per class (training and test set combined) that was collected by LeCun and colleagues [[1]](https://paperpile.com/c/htO4HB/eXc6Y), and is one of the most commonly used benchmarks to evaluate the performance of deep learning models.

#### S1.2.2 Fashion MNIST dataset

The Fashion MNIST dataset [[2]](https://paperpile.com/c/htO4HB/oXfRs) consists of 60,000 samples of 28x28 grayscale images (i.e., the dimensionality of 784 pixels) of 10 clothing categories (i.e., shirt, shoes, etc) with a total of 7000 images per class (training and test set combined) [[2]](https://paperpile.com/c/htO4HB/oXfRs).

#### S1.2.3 fMRI dataset

##### S1.2.3.1 Participants & task

The fMRI dataset used here was collected previously for several separate projects and was partially reported previously by Taschereau-Dumouchel and colleagues [[3]](https://paperpile.com/c/htO4HB/bMPAD). The dataset we used here contained 60 usable subjects’ whole-brain data; one subject’s data produced wildly unstable outcomes across all metrics tested here (see **Methods 4.4**, main text) and so was excluded from the final analysis, and an additional 10 subjects had previously been collected with a different imaging sequence and are not included here. From this dataset, we therefore examined 59 healthy human participants who had viewed 3600 images from 40 categories (90 exemplars of each category, including 30 categories of animals [dogs, cats, snakes, etc.] and 10 categories of man-made objects [keys, chairs, airplanes, etc.]) while whole-brain BOLD responses were acquired. The images were shown for 0.98s each in mini-blocks (chunks) of 2, 3, 4, or 6 images of each category displayed sequentially, and the participants were asked to press a button whenever the category of images was changed. The data was collected from each participant in six runs with short breaks in approximately one hour while they were in the scanner. Each image was on screen for a duration of 0.98 s. The voxel activities of the ventral temporal cortex (VTC) were used as input to the model in order to find the latest features represented by VTC. See the methods reported by Taschereau-Dumouchel and colleagues [[3]](https://paperpile.com/c/htO4HB/bMPAD) for further details, including information on informed consent and ethics approval for this previously-existing dataset.

##### S1.2.3.2 Image acquisition & preprocessing

The functional imaging data acquisition and preprocessing procedures for this existing dataset are previously described elsewhere [[3]](https://paperpile.com/c/htO4HB/bMPAD), but are included here for completeness. Participants in the database were scanned at one of two 3T MRI scanners (Siemens Prisma and Verio) with a head coil. Whole brain functional data were acquired in 33 contiguous slices (TR = 2000 ms, TE =30 ms, voxel size = 3 × 3 × 3.5 mm 3, field-of-view = 192 x 192 mm, matrix size = 64 x 64, slice thickness = 3.5 mm, 0 mm slice gap, flip angle = 80 deg) oriented parallel to the AC-PC plane. High-resolution T1-weighted structural MR images (MP-RAGE sequence; 256 slices, TR = 2250 ms, TE = 3.06 ms, 5 voxel size = 1 × 1 × 1 mm 3, field-of-view= 256 x 256 mm, matrix size = 256 x 256, slice thickness = 1 mm, 0 mm slice gap, TI = 900 ms, flip angle = 9 deg.) were also obtained. Functional images were preprocessed using standard procedures, including slice timing correction, motion correction, realignment to the first functional image, and coregistration to the structural scan using SPM 12 (Statistical Parametric Mapping; [www.fil.ion.ucl.ac.uk/spm](http://www.fil.ion.ucl.ac.uk/spm)). The anatomical mask of the target region of interest, VTC (fusiform, lingual/parahippocampal, and inferior temporal cortex), was selected using the Freesurfer (<http://surfer.nmr.mgh.harvard.edu/>) automated gray matter segmentation combining the ROI labels of *fusiform*, *inferior temporal*, *lingual*, and *parahippocampal*.

Voxels in this combined ventral temporal ROI were detrended and then deconvolved using the least-square separate approach [[4,5]](https://paperpile.com/c/htO4HB/4MTyN+b9KBX). This method creates an iterative general linear model for each trial individually, such that the design matrix contains one parameter modeling the current trial, and two parameters modeling all other trials in the design (e.g., even- and odd-numbered trials). This standardized method allows deconvolution of each trial in a rapid-event related design such as this one to obtain parameter estimates for each individual trial. This process results in a N_VTC,S_ x 3600 images (90 exemplars of each category) timeseries, where N_VTC,S_ refers to the number of voxels in the combined VTC ROI for each subject S; in this dataset N_VTC,S_ ranged from ~40 to 55 MB in CSV format. This timeseries formed the dataset used in this project.

### S1.3 Models

#### S1.3.1 Task-Relevant Autoencoder via Classifier Enhancement (TRACE) model

The base of the TRACE architecture consists of input and output layers with dimensionality equal to the size of the datasets (784 for MNIST and Fashion MNIST, and 1726-3078 for fMRI), plus a single hidden layer with 1000 units in each of the encoding and decoding sections.

##### S1.3.1.1 Inputs

To facilitate comparisons across the three datasets tested and to facilitate faster model convergence, we standardized inputs by scaling their values: MNIST and Fashion MNIST inputs were scaled to take on values between 0 and 1 by dividing all values by 255, and fMRI inputs (parameter estimates from the single-trial deconvolution described above) were standardized by z-scoring because these parameter estimates can take on arbitrary real numbers without bound. To prevent leakage of any task-relevant information from test sets to the training set, the scaling factors were determined only on the training set, and then the same scaling parameters were used to scale the test sets.

##### S1.3.1.2 Activation functions

For the hidden layers, we used the hyperbolic tangent as the activation function in order to discover more complex nonlinear patterns in the data, as this function was reported previously to be more sensitive in capturing detailed and local information to represent the data with lower dimensions [[6]](https://paperpile.com/c/htO4HB/9ACIb). For the bottleneck layer of the network, we selected the linear activation function (i.e., no activation function) because in initial explorations we found that using a linear function resulted in discovering more task-relevant features in comparison to other activation functions, such as hyperbolic tangent, rectified linear unit (ReLu) and sigmoid, since features of the bottleneck layer under the linear function had higher accuracy in decoding the categories (e.g. in the case of MNIST dataset and with 2 dimensions in the bottleneck the accuracy increases by 15 percent; full data not shown). For the final decoding layer, a linear activation function was also chosen because the fMRI data are unitless and take arbitrary numbers and therefore are not confined to be within a specific boundary. Using a linear function allows the reconstructing layer to assign any value that minimizes the difference between output and input of the autoencoder, unlike most other typical activation functions (e.g., sigmoid, Tanh, etc) which usually have a confined output value hence those are not good choices to reconstruct fMRI data.

##### S1.3.1.3 Loss function minimization

To minimize TRACE’s objective function (**Equation 3**, main text), the Adam [[7]](https://paperpile.com/c/htO4HB/PGTge) implementation of stochastic gradient descent (SGD) was used and the learning rate was chosen to be 1e-4 and the batch size was set to 32. To prevent overfitting, the dropout technique was used for regularization with a dropout rate of 0.1.

#### S1.3.2 Standard autoencoder (AE) model

The standard AE model provides a baseline benchmark of model behavior in all datasets tested. It is therefore identical in form and implementation to TRACE with the exception of its objective function, which is simply the mean squared error (MSE):

$L_{R} = \frac{1}{m\times n} \sum_{i=1}^{m} \sum_{j=1}^{n} ( \hat{X}_{ij}- X_{ij})^{2}$ (S1)

where $X$ is the input with $m$ samples and $n$ input-dimensions, and $\hat{X}$ is the reconstruction of the input.

#### S1.3.3 Variational autoencoder (VAE)

The variational autoencoder used here is identical to AE in terms of the architecture and hyperparameters. The only difference is that unlike AE and TRACE we used a softmax function at the last layer in the case of MNIST and Fashion MNIST datasets. We did this because the network performed very poorly when we used a linear function (i.e., no activation function). The loss function was defined as follows:

$L_{VAE} =L_{R} + L_{KL}$ (S2)

where $L_{R}$ is the reconstruction loss and $L_{KL}$, Kullback–Leibler (KL) divergence, is to calculate the distance between the encoder distribution $q(z|x)$ and a prior distribution $p(z)$ which was chosen to be a standard gaussian distribution. Therefore the $L_{KL}$ becomes

$L_{KL}= -0.5\sum_{z} [1+log\sigma_{z}^{2}-\mu_{z}^{2}-\sigma_{z}^{2}]$ (S3)

where $\mu_{z}$and $\sigma_{z}$are mean and standard deviation of the latent space in the bottleneck.

#### S1.3.4 Details of dataset size and data truncation

The MNIST and Fashion MNIST datasets, each using 60,000 training and 10,000 test samples, are popular choices in benchmarking deep learning models. Having a high ratio of training samples for the number of data dimensions in these datasets (i.e., 60,000/748 = 76.5) makes them good candidates even for very deep neural networks. However, verifying the advantage of a model on these huge dataset is not necessarily applicable to dataset with much smaller sample to input-dimension size ratio (e.g., the real-world fMRI dataset we used here with approximate samples-to-input-dimensions ratio of ~1.5), as such a ratio might lead to overfitting and thus poor predictive capacity. To ensure that TRACE is powerful not only when the samples-to-input-dimensions ratio is large but also when available data is much sparser (such as in biological datasets that often suffer from small sample size), we truncated the MNIST and fashion MNIST datasets to explore how TRACE behaves under increasing data truncation.

To accomplish this, we trained all models at 10, 30, 50, 70, 90, 95, and 98 percent of data truncation at the optimal bottleneck dimensionality (i.e., d=2) for both MNIST and Fashion MNIST. Reducing the number of training samples to 95 and 98 percent decreases the samples-to-input-dimensions ratio such that it is approximately the same as the fMRI dataset also used here. The training set at each level of data truncation was the same for all models. To prevent overfitting caused by the limited sample size in the truncation analysis, we adjusted the stopping rule to stop the training when there is no improvement in the optimization process for 20 epochs, with a maximum training of 300 epochs. We then evaluated the behavior of all four outcome metrics under all levels of data truncation by using the conventional hold-out test set in the MNIST and Fashion MNIST (i.e., 10,000 sample test set).

In the case of the fMRI dataset, we performed the training on 2700 training samples and tested the trained models on 900 held-out test samples. At the extreme level of 98% data truncation in the MNIST and Fashion MNIST datasets, the sample size is reduced to 1200 exemplars with each exemplar having 784 input-dimensions. Therefore, the ratio of samples to input-dimensions is about 1.5 which is comparable to sample-to-input-dimension ratio of fMRI dataset (i.e., ~1.5).

#### S1.3.4 Implementation details for models

In order to tune the hyperparameter 𝛼 (**Equation 3**, main text) to control for the contribution of the logistic classifier to the final objective function of TRACE, we chose different values for 𝛼 (i.e., 0, 0.01, 0.1, 0.2, 0.5, 0.9, 1) for the bottleneck dimensionality of d=2. We chose 𝛼 as 0.01 since it seemed it is the optimum point for the reconstruction and classification trade-off. Surprisingly, at 𝛼=0.01 reconstruction loss was actually lower than 𝛼=0 and the total loss was found to be almost the same as 𝛼=0.


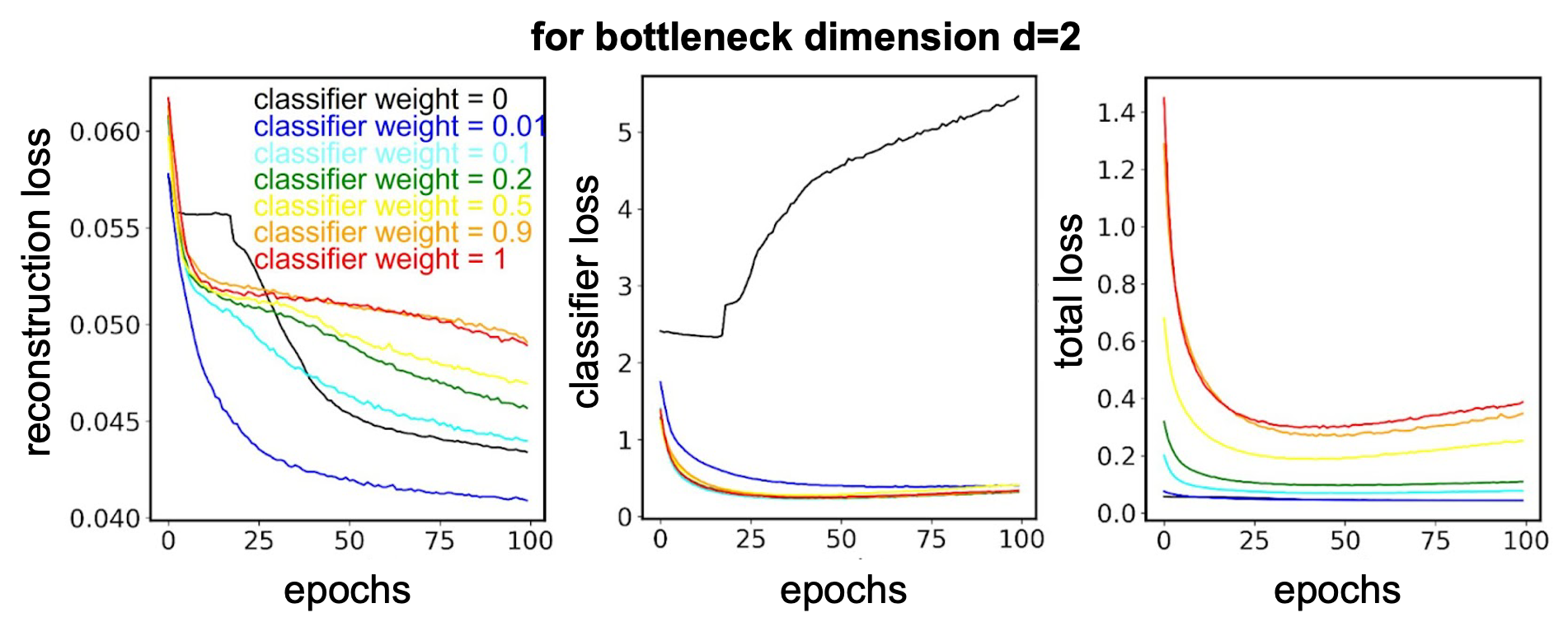


**Figure S1.** **Loss functions for the MNIST dataset as a function of training epochs for bottleneck dimensionality d=2, for different levels of the hyperparameter 𝛼.** We selected 𝛼=0.01 for all results presented in the main text because it showed the fastest convergence and achieved the smallest total loss (bottom panel) when training was complete.

For all datasets and all networks we used the same architecture and hyperparameter values. To implement these networks, we used the Keras functional API [[8]](https://paperpile.com/c/htO4HB/rw2Va) with TensorFlow [[9]](https://paperpile.com/c/htO4HB/cagr4) backend, using available GPUs in Google Colab Pro and 12.7 GB of RAM.

#### S1.3.5 Implementation details for quantitative comparison metrics

For bottleneck and reconstruction classifier accuracy (and their input-based equivalents), the hyperparameter $\lambda$ was set to 0.007 which was manually tuned to maximize the classification accuracy. Learning rate was set to 0.5e-4. Logistic classifiers were trained with L2 regularization. Using the same train/test folds as for training all models, we trained the classifiers for 30 epochs for MNIST and Fashion MNIST and 300 epochs for fMRI.

### S1.4 Model fitting practical details

In order to meaningfully compare behaviors between TRACE and the other models (AE, VAE, and PCA), it is important to determine that both models can both be adequately fit to each of our datasets. Using available GPU processors in Google Colab Pro, it took about 61 minutes to fit the AE model to the MNIST training dataset (60,000 labeled samples), and about the same time for Fashion MNIST training dataset (60,000 labeled samples), and 21 minutes on average for each human subject in the fMRI dataset (3600 labeled samples of VTC) and for all 14 dimensionalities of bottleneck.

## S2 Comprehensive discussion of model performance

In the main text, we present and discuss the results from bottleneck dimensionalities between 2 - 150 for MNIST and Fashion MNIST, because at d>150 the quantitative metrics (see Methods) tend to asymptote. **Figure S2** presents the four metrics at bottleneck dimensions up to 784 as a comprehensive demonstration of this behavior. **Table S1** provides the bottleneck dimensionality at which each metric is maximized.


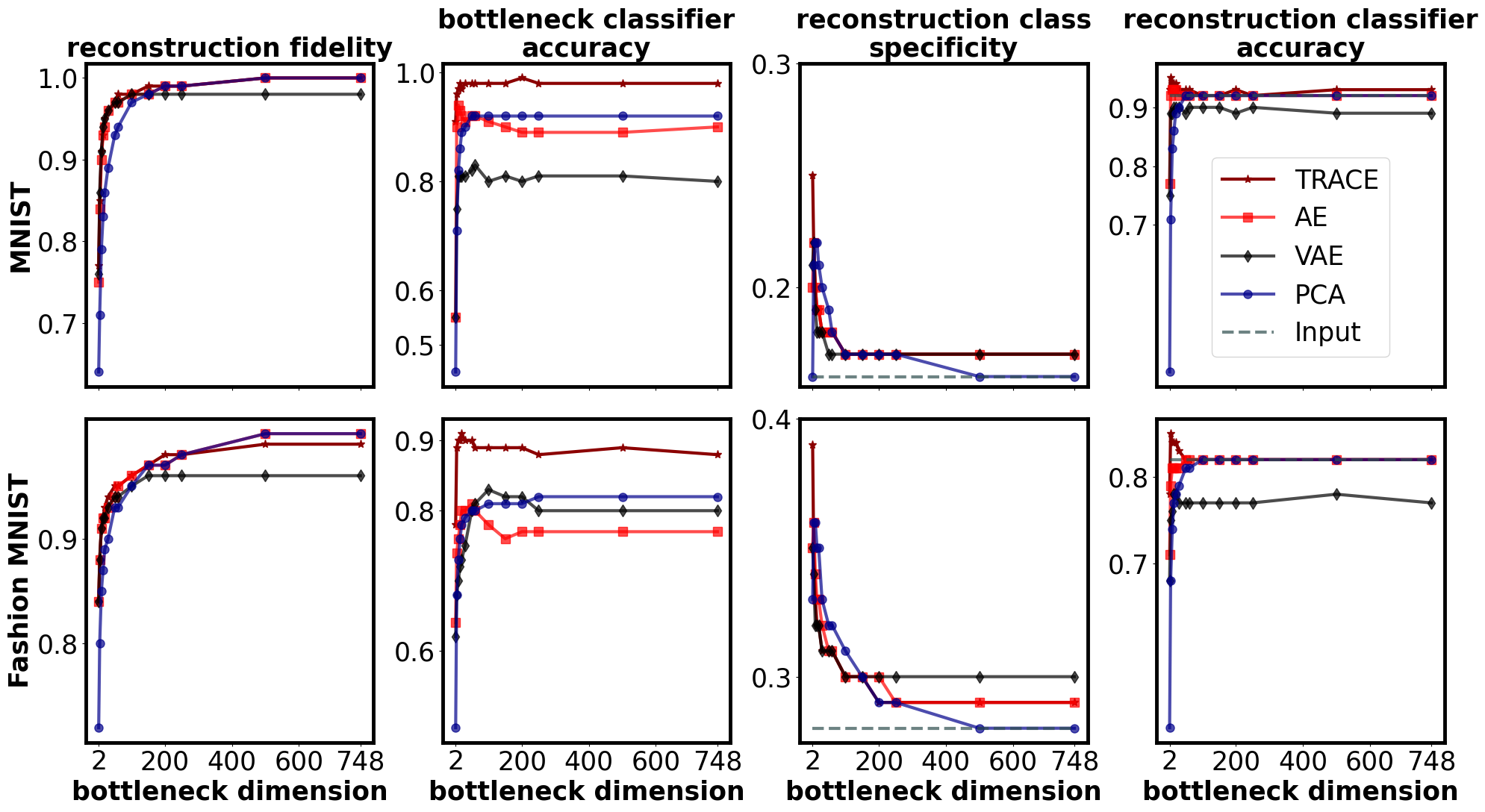


**Figure S2.** **Quantitative comparison between TRACE and AE on the four outcome metrics, for all three datasets (MNIST, Fashion MNIST, and fMRI) for bottleneck dimensionalities between 2 and 784.** Here we partially replicate **Figure 6** from the main text to show comparisons directly between MNIST, Fashion MNIST, and fMRI data. As in the main text, dashed lines represent the input reconstruction class specificity and the input reconstruction classifier accuracy, respectively.

|  | |  | **MNIST** | **Fashion MNIST** | **fMRI** |
| --- | --- | --- | --- | --- | --- |
| **1** | **Reconstruction fidelity** | **TRACE** | 500 | No peak | No peak |
|  |  | **AE** | 500 | 500 | No peak |
|  |  | **VAE** | No peak | No peak | No peak |
|  |  | **PCA** | 500 | 500 | No peak |
| **2** | **Bottleneck classifier accuracy** | **TRACE** | No peak | 20 | No peak |
|  |  | **AE** | 10 | 50 | No peak |
|  |  | **VAE** | 60 | 100 | No peak |
|  |  | **PCA** | No peak | No peak | No peak |
| **3** | **Reconstruction class specificity** | **TRACE** | 2 | 2 | 30 |
|  |  | **AE** | 5 | 5 | 50 |
|  |  | **VAE** | 5 | 2 | 50 |
|  |  | **PCA** | 10 | 5 | 50 |
| **4** | **Reconstruction classifier accuracy** | **TRACE** | 5 | 5 | No peak |
|  |  | **AE** | 10 | No peak | No peak |
|  |  | **VAE** | 15 | 15 | No peak |
|  |  | **PCA** | No peak | No peak | No peak |

**Table S1. Bottleneck dimensionality at which each outcome metric is maximized.**

|  |  |  | **MNIST** | **Fashion MNIST** | **fMRI** |
| --- | --- | --- | --- | --- | --- |
| **1** | **Reconstruction fidelity** | **TRACE** | 0.77 | 0.84 | 0.73 |
|  |  | **AE** | 0.75 | 0.84 | 0.73 |
|  |  | **VAE** | 0.76 | 0.84 | 0.67 |
|  |  | **PCA** | 0.64 | 0.72 | 0.65 |
| **2** | **Bottleneck classifier accuracy** | **TRACE** | 0.91 | 0.78 | 0.16 |
|  |  | **AE** | 0.55 | 0.64 | 0.07 |
|  |  | **VAE** | 0.55 | 0.62 | 0.07 |
|  |  | **PCA** | 0.45 | 0.49 | 0.03 |
| **3** | **Reconstruction class specificity** | **TRACE** | 0.25 | 0.39 | 0.05 |
|  |  | **AE** | 0.2 | 0.35 | 0.029 |
|  |  | **VAE** | 0.21 | 0.35 | 0.034 |
|  |  | **PCA** | 0.16 | 0.33 | 0.034 |
| **4** | **Reconstruction classifier accuracy** | **TRACE** | 0.93 | 0.78 | 0.4 |
|  |  | **AE** | 0.77 | 0.71 | 0.32 |
|  |  | **VAE** | 0.75 | 0.68 | 0.22 |
|  |  | **PCA** | 0.45 | 0.51 | 0.17 |

**Table S2. Performance at bottleneck dimensionality d=2 for MNIST and Fashion MNIST, and d=30 for fMRI.**

|  | **Reconstruction fidelity** | **Bottleneck classifier accuracy** | **Reconstruction class specificity** | **Reconstruction classifier accuracy** |
| --- | --- | --- | --- | --- |
| **Main effect** | F(3,147)=7927, p<1e-3 | F(3,147)=579,  p<1e-3 | F(3,147)=1181, p<1e-3 | F(3,147)=2257, p<1e-3 |
| **TRACE vs AE** | t(49)=20.1, p<1e-3 | t(49)=11.03, p<1e-3 | t(49)=22.65, p<1e-3 | t(49)=23.8,  p<1e-3 |
| **TRACE vs VAE** | t(49)=74.8, p<1e-3 | t(49)=34.26, p<1e-3 | t(49)=35.17, p<1e-3 | t(49)=53.3,  p<1e-3 |
| **TRACE vs PCA** | t(49)=173.8, p<1e-3 | t(49)=35.07, p<1e-3 | t(49)=74.07, p<1e-3 | t(49)=70.4,  p<1e-3 |

**Table S3. Results of ANOVAs and planned pairwise contrasts comparing TRACE to all other models at 98% data truncation for the MNIST and Fashion MNIST datasets, across all four metrics.**

|  | **Reconstruction fidelity** | **Bottleneck classifier accuracy** | **Reconstruction class specificity** | **Reconstruction classifier accuracy** |
| --- | --- | --- | --- | --- |
| **Main effect** | F(3,171)=83.1, p<1e-3 | F(3,171)=356, p<1e-3 | F(3,171)=111, p<1e-3 | F(3,171)=433, p<1e-3 |
| **TRACE vs AE** | t(57)=-0.798, p=0.855 | t(57)=17.143, p<1e-3 | t(57)=18.822, p<1e-3 | t(57)=10.61,  p<1e-3 |
| **TRACE vs VAE** | t(57)=7.682, p<1e-3 | t(57)=16.826, p<1e-3 | t(57)=11.836, p<1e-3 | t(57)=23.45,  p<1e-3 |
| **TRACE vs PCA** | t(57)=9.825, p<1e-3 | t(57)=28.663, p<1e-3 | t(57)=10.055, p<1e-3 | t(57)=34.32,  p<1e-3 |

**Table S4. Results of ANOVAs and planned pairwise contrasts comparing TRACE to all other models for the fMRI dataset, across all four metrics.**

Out of desire to explore TRACE’s behavior for the fMRI dataset in particular, we also computed Euclidean distance-based representational dissimilarity matrices for the raw input, and bottleneck representations and reconstructions at optimal bottleneck dimensionality. When arranging the animals and objects themselves in a logical order (insects versus other animals versus man-made objects), both the bottleneck and reconstruction showed clearer clusters than the raw input space (**Figure S3**).


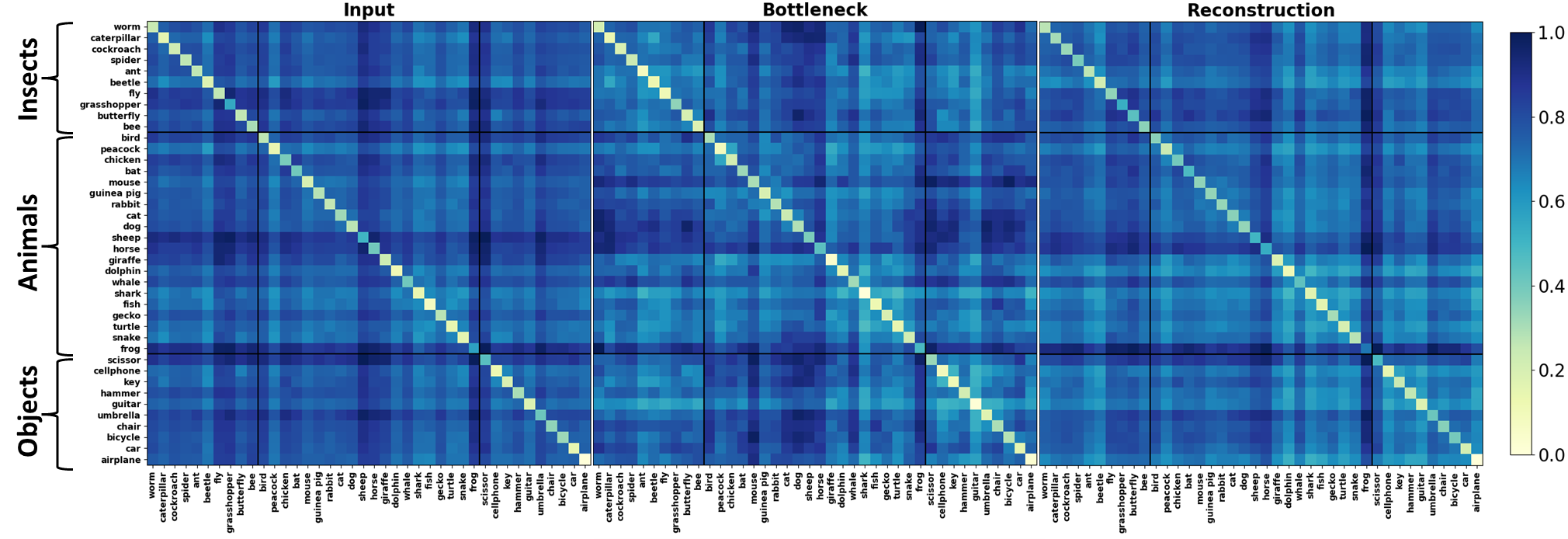


**Figure S3. Representational dissimilarity matrices for input, bottleneck, and reconstruction in the fMRI dataset.** Average Euclidean distances ($D\left( i,j \right)= \frac{1}{S\times M}\sum_{s=1}^{S} \sum_{m=1}^{M} d_{s,m}(i,j))$where $S$ is the number of subjects, $M$ is the number of pairs between categories $i$ and $j$, and $d$ is the Euclidean distance between two pairs of exemplars; Euclidean distances are normalized within each plot such that the maximum distance is rescaled to 1 and the minimum distance rescaled to 0) between all pairs of objects (within- and between-classes), and across 58 subjects (one subject was excluded as an outlier). Clusters are visible in the bottleneck and reconstruction that are not visible in the input.

## S3 Comparison of reconstructions with other models

In addition to the three other benchmark models (i.e., AE, VAE, and PCA), we compared TRACE’s reconstructions of MNIST and Fashion MNIST with reconstructions achieved with a conditional generative adversarial network (cGAN) [[10]](https://paperpile.com/c/htO4HB/4F6Na). As illustrated in **Figure S4**, the cGAN demonstrates strong performance when abundant data is available (e.g., 60,000 exemplars). However, the performance of cGAN significantly deteriorates in the face of data sparsity as depicted in **Figure S4** (e.g., 1200 exemplars). Therefore, using cGAN may not be a practical choice in the case of fMRI datasets which typically consist of only a few hundred exemplars.

We also benchmarked TRACE against a denoising autoencoder (DAE) [[11]](https://paperpile.com/c/htO4HB/Lsb0O). However, we noticed that the DAE is primarily successful in eliminating the noise that we introduce to the input during training, and it fails to perform well when the structure of the noise is changed or unknown (which is always the case in fMRI datasets). Furthermore, DAE exhibited significant instability across some of the outcome metrics, leading us to opt against further exploration of this model or its inclusion in this manuscript.


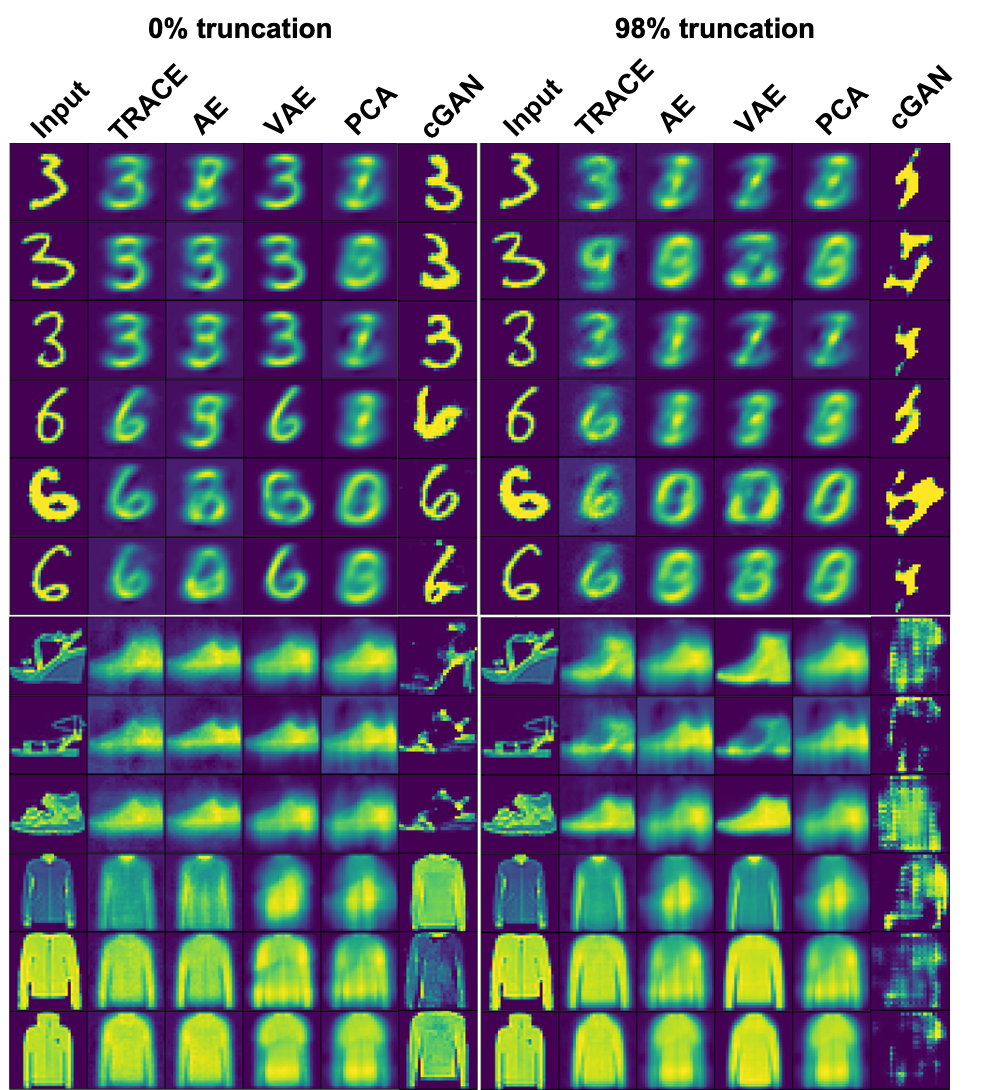


**Figure S4. Reconstructions from all four models tested in the main text, plus a conditional generative adversarial network (cGAN).** The cGAN performs quite well, according to visual inspection, when trained on 100% of the MNIST or Fashion MNIST datasets. However, at 98% truncation (trained on only 2% of the data), the cGAN fails dramatically.

#

# Supplementary References

1. [LeCun Y, Cortes C, Burges C. MNIST handwritten digit database, 1998. URL http://www research att com/\ yann/ocr/mnist. 1998.](http://paperpile.com/b/htO4HB/eXc6Y)

2. [Xiao H, Rasul K, Vollgraf R. Fashion-MNIST: a Novel Image Dataset for Benchmarking Machine Learning Algorithms. arXiv [cs.LG]. 2017. Available:](http://paperpile.com/b/htO4HB/oXfRs) <http://arxiv.org/abs/1708.07747>

3. [Taschereau-Dumouchel V, Cortese A, Chiba T, Knotts JD, Kawato M, Lau H. Towards an unconscious neural reinforcement intervention for common fears. Proc Natl Acad Sci U S A. 2018;115: 3470–3475.](http://paperpile.com/b/htO4HB/bMPAD)

4. [Mumford JA, Turner BO, Ashby FG, Poldrack RA. Deconvolving BOLD activation in event-related designs for multivoxel pattern classification analyses. Neuroimage. 2012;59: 2636–2643.](http://paperpile.com/b/htO4HB/4MTyN)

5. [Turner BO, Mumford JA, Poldrack RA, Ashby FG. Spatiotemporal activity estimation for multivoxel pattern analysis with rapid event-related designs. Neuroimage. 2012;62: 1429–1438.](http://paperpile.com/b/htO4HB/b9KBX)

6. [Ng WWY, Zeng G, Zhang J, Yeung DS, Pedrycz W. Dual autoencoders features for imbalance classification problem. Pattern Recognit. 2016;60: 875–889.](http://paperpile.com/b/htO4HB/9ACIb)

7. [Kingma DP, Ba J. Adam: A Method for Stochastic Optimization. arXiv [cs.LG]. 2014. Available:](http://paperpile.com/b/htO4HB/PGTge) <http://arxiv.org/abs/1412.6980>

8. [Chollet F, Others. Keras documentation. keras io. 2015;33.](http://paperpile.com/b/htO4HB/rw2Va)

9. [Abadi M, Agarwal A, Barham P, Brevdo E, Chen Z, Citro C, et al. TensorFlow: Large-Scale Machine Learning on Heterogeneous Distributed Systems. arXiv [cs.DC]. 2016. Available:](http://paperpile.com/b/htO4HB/cagr4) <http://arxiv.org/abs/1603.04467>

10. [Mirza M, Osindero S. Conditional Generative Adversarial Nets. arXiv [cs.LG]. 2014. Available:](http://paperpile.com/b/htO4HB/4F6Na) <http://arxiv.org/abs/1411.1784>

11. [Vincent P. Stacked denoising autoencoders: Learning useful representations in a deep network with a local denoising criterion. Journal of Machine Learning Research. 2010;11: 3371–3408.](http://paperpile.com/b/htO4HB/Lsb0O)
